# Standardized Photobiomodulation Dosimetry Targeting the Base of Calvarial Critical-Sized Defects for Bone Regeneration: A Preclinical RCT Comparing Flattop vs. Gaussian Beam Profiles, with or Without Bio-Oss^®^

**DOI:** 10.3390/jfb17030125

**Published:** 2026-03-04

**Authors:** Reem Hanna, Wayne Selting, Vincenzo Cuteri, Giacomo Rossi, Alessandro Bosco, Laura Emionite, Michele Cilli, Emanuela Marcenaro, Federico Rebaudi, Marco Greppi, Stefano Benedicenti

**Affiliations:** 1 Department of Restorative Dental Sciences, Eastman Dental Institute, Medical Faculty, University College London (UCL), 21 University Street, London WC1E 6DE, UK; 2 Department Head and Neck Academic Centre, Integrated Research, Division of Surgery and Intervention Sciences, Medical Faculty, University College London (UCL), 43-45 Foley Street, London W1W 7TY, UK; 3 Dental Faculty, Royal College of Surgeons Ireland, 121-122 St Stephen’s Green, Dublin 2, D02 H903 Dublin, Ireland; 4 Department of Surgical Sciences and Integrated Diagnostics, University of Genoa, 16132 Genoa, Italy; 5 School of Biosciences and Veterinary Medicine, University of Camerino, Via Circonvallazione, 93/95, Matelica, 62024 Camerino, Italy; 6 Bosco Ottico S.r.l, Via Ceradelli, 10, Castel Rozzone, 24040 Bergamo, Italy; 7 Animal Facility, Istituto di Ricovero e Cura a Carattere Scientifico (IRCCS) Ospedale Policlinico San Martino, Largo Rosanna Benzi 10, 16132 Genoa, Italy; 8 Department of Experimental Medicine (DIMES), University of Genoa, Viale Benedetto XV, 1, 16132 Genoa, Italy; 9 IRCCS Ospedale Policlinico San Martino, Largo Rosanna Benzi, 10, 16132 Genoa, Italy

**Keywords:** 980 nm, Bio-Oss^®^, bone regeneration, calvarial defect, flattop beam profile, gaussian profile, histology, immunohistochemistry, photobiomodulation, osteogenesis

## Abstract

Photobiomodulation (PBM) has shown promising potential to enhance bone regeneration; however, its optimal delivery parameters and interactions with osteoconductive scaffolds remain insufficiently defined. This preclinical study is the first to incorporate a pilot dosimetry evaluation to standardize 980-nm PBM delivery and ensure that effective irradiance reached the target surface of critical-size calvarial defects in mice. The primary aim was to evaluate the effectiveness of this novel 980-nm PBM protocol delivered using either flat-top (FT) or standard Gaussian (ST) handpieces in enhancing bone regeneration in critical-size defects (CSDs), both with and without Bio-Oss^®^ grafting. A total of 120 adult mice were allocated into twelve experimental groups (*n* = 10 per group): untreated (control), Bio-Oss^®^ alone, PBM alone, and PBM combined with Bio-Oss^®^, using either FT or ST handpieces, and evaluated at 30 and 60 days. Animals received 980 nm irradiation at 0.6 W (nominal power output–set on laser interface) in continuous-wave mode for 60 s, three times per week, for two consecutive weeks. Pilot dosimetry included power meter measurements to determine the therapeutic power reaching the defect surface area and temperature monitoring to ensure safe energy delivery. The dosimetry study demonstrated that, after accounting for the optical properties of mouse shaved skin and the Bio-Oss^®^ graft covered with Bio-Gide^®^ membrane, the effective irradiance reaching the base of the defect surface area was 1.131 W/cm^2^ for the FT handpiece and 0.413 W/cm^2^ for the ST handpiece. This dose was sufficient to induce significant regenerative effects. Histological, Masson’s trichrome, and immunohistochemical analyses for Runx2, OCN, GLI1, CD34, and CTSK were performed to characterize early and late osteogenic events. The combination of PBM and Bio-Oss^®^ significantly accelerated bone regeneration compared with PBM alone, with the FT handpiece producing the most uniform and advanced osteogenesis. PBM enhanced progenitor activation, osteoblast differentiation, angiogenesis, matrix deposition, and late-stage remodeling, demonstrating a synergistic effect with the scaffold, whereas Bio-Oss^®^ alone or defect alone showed limited early regenerative potential. These findings highlight the effectiveness of this novel standardized PBM dosimetry and uniform beam profile (FT), supporting their use as a foundation for future randomized controlled trials in craniofacial bone repair.

## 1. Introduction

Regenerative management of critical-sized bone tissue defects resulting from trauma, tumor resection, congenital malformations, or systemic diseases such as osteoporosis, diabetes mellitus, and rheumatoid arthritis remains a major clinical challenge [1,2]. These defects are designated as critical-sized because they exceed the threshold for spontaneous healing. Bone ingrowth from the defect margins is insufficient to bridge the defect, and without intervention, a permanent defect persists.

The clinical management of such defects is complex and often requires surgical intervention using bone grafting materials. However, current treatment strategies are associated with long healing times, potential complications, and increased healthcare costs. Despite advances in biomaterials with osteoinductive properties, such as bioglasses, polymeric scaffolds, and composite materials, significant challenges remain in achieving optimal vascularization and robust host integration [3,4].

One widely used xenograft material is Bio-Oss^®^, a deproteinized bovine bone mineral. It is commonly applied in the field of oral and maxillofacial surgery and is valued for its osteoconductive and biocompatible properties [4,5,6,7]. Its porous architecture facilitates clot stabilization and supports new bone ingrowth [8,9,10]. However, Bio-Oss^®^ is biologically inert and undergoes slow resorption, which may limit its regenerative capacity in time-sensitive clinical scenarios [11,12]. In animal models of calvarial bone defects, Bio-Oss^®^ demonstrated approximately 25% greater dimensional stability compared with autogenous bone [13]. Moreover, its hydrophilic properties promote rapid and complete hydration [13]. It provides standardized outcomes in in vivo animal studies [13,14].

### 1.1. Rationale for Photobiomodulation Modality

Photobiomodulation (PBM), as a non-invasive approach, can be a potential therapeutic modality to stimulate tissue repair, modulate inflammation, and enhance cellular activity [15,16]. PBM modulates cellular behavior by delivering photonic energy to intracellular chromophores, particularly within the mitochondria, resulting in increased ATP production, regulation of reactive oxygen species (ROS), and activation of transcription factors. These effects enhance protein synthesis, Ca^2+^ uptake, and cell proliferation, which are key processes in bone healing [17,18,19,20].

Growth factor release, angiogenesis, and modulation of inflammatory pathways are also stimulated by PBM, thereby supporting enhanced tissue repair and pain reduction [21,22]. Infrared diode lasers, in particular, have shown promising effects on bone healing in both healthy and diseased bone, especially during early osseointegration phases [23,24,25].

Animal studies evaluating PBM in combination with bone grafts have shown accelerated healing and improved bone-implant contact [26,27]. Nonetheless, despite encouraging findings, the mechanisms by which PBM influences bone tissue and biomaterials remain incompletely understood.

### 1.2. Challenges in PBM Clinical Translation

The clinical translation of PBM has been significantly hindered by variability in dosimetry parameters such as wavelength, irradiance, emission mode, fluence, treatment duration, and beam profile. In particular, inconsistent irradiance levels reaching the target surface area have made it difficult to compare studies or establish standardized treatment protocols [16,26]. Although PBM has demonstrated potential therapeutic benefits across various clinical applications, clearly defined guidelines regarding therapeutic irradiance at the target tissue are still lacking. This parameter is critical for achieving reproducible outcomes and standardizing PBM dosimetry.

To address this limitation, the use of a Flattop (FT) handpiece has been proposed as a strategy to improve therapeutic consistency and standardization. This innovation may enhance reproducibility and optimize clinical outcomes in PBM therapy [27,28,29].

### 1.3. Knowledge Gaps and Rationale for the Present Study

Although PBM has shown promising effects on bone regeneration, its effects in the presence of xenograft materials such as Bio-Oss^®^ remain under-investigated. It is unclear whether PBM exerts synergistic effects when combined with such biomaterial and how these interactions influence bone healing at the cellular and molecular levels [27].

Our group and others have shown that PBM can stimulate osteoblastic cell lines in vitro under controlled parameters [28,29]; however, in vivo (animal and human) validation is still required. In vitro studies have shown stimulatory effects of PBM on osteoblast-like cells, including enhanced proliferation, differentiation, and mitochondrial activation. However, its precise mechanism of action remains incompletely understood, particularly in the context of biomaterial-based bone regeneration [30,31].

Additionally, interactions between light and certain biomaterials may reduce the therapeutic efficacy of PBM. This was demonstrated in a preliminary study conducted by Varela et al., 2023 [32], which reported that collagen membranes can attenuate the laser light and potentially diminish the biological effects of PBM.

Furthermore, most existing studies are characterized by non-standardized parameters and substantial variability in dosimetry, laser type, and beam delivery profiles. These inconsistencies limit the clinical applicability of PBM in bone healing [33]. There is substantial variability in experimental protocols. This includes differences in wavelength, power output, energy delivered to the target surface area, irradiance, and energy density. In addition, power meters are often not used to measure the actual therapeutic irradiance reaching the target surface. As a result, establishing evidence-based therapeutic dosimetry remains challenging [34,35,36].

A recent systematic review by Moscatel et al., 2025 [37] highlighted the lack of standardized dosimetry parameters in PBM research. The most frequently reported energy densities were 4, 16, and 30 J/cm^2^, whereas higher doses ranging from 210 to 354 J/cm^2^ were less commonly applied. This broad range of parameters further complicates interpretation and reproducibility.

Similarly, a comprehensive systematic review by Hanna et al., 2021 [38], which analyzed 38 in vivo animal studies involving PBM with or without bone grafts, reported generally positive effects on healing. However, the authors emphasized that the lack of PBM dosimetry standardization limited meta-analysis and cross-study comparison.

### 1.4. Aim and Objectives of the Study

This translational in vivo study aims to evaluate the efficacy of a novel 980 nm PBM laser protocol, delivered via FT and ST handpieces, for enhancing bone regeneration in critical-size defects (CSDs) with and without Bio-Oss^®^ grafting. Specifically, the study aimed to address the following research questions:

1. PBM dosimetry accuracy: Does the novel 980 nm laser protocol deliver accurate and reproducible energy reaching the target tissue in vivo?
2. Beam profile effect: Does the FT beam profile enhance osteogenesis compared to the ST profile and untreated controls?
3. PBM–biomaterial synergy: Is there a synergistic effect between 980 nm PBM and Bio-Oss^®^ in promoting bone regeneration?

The Objectives of the study are listed below:

1. To validate a novel PBM dosimetry protocol for clinical translational research;
2. To compare the effects of FT vs. ST beam profiles on bone regeneration in CSD;
3. To assess whether combining PBM with Bio-Oss^®^ improves osteogenesis and offers synergetic effects;
4. To understand the mechanism of the PBM interaction with the Bio-Oss^®^ grafting;
5. To assess osteogenic activity and bone formation at 30-days and 60-days post-treatment utilizing accepted analysis methods:
  - Histological analysis: evaluating new bone formation and tissue organization.
  - Immunohistochemical analysis of molecular biomarkers:
    - CD34: vascular presence/vascularization);
    - Gli1: early osteoprogenitor/stem cell activations;
    - Runx2: early-mid osteoblast differentiation;
    - Osteocalcin (OCN): late osteoblast maturation and matrix mineralization;
    - Cathepsin K (CTSK): osteoclast-mediated bone remodeling.

## 2. Materials and Methods

### 2.1. Study Design and Ethical Approval

This study was a multi-arm, randomized, double-blind, comparative, interventional in vivo animal trial. The experimental protocol was approved by the Ethical Committee of the University of Camerino, Italy, under protocol number CEEPA 009/2016, authorization number 518/2020-PR (Reference: Prot. ID580.30) on 22 May 2020.

The study was conducted in accordance with the ARRIVE (Animal Research: Reporting of In Vivo Experiments) guidelines 2.0 and the EU Directive 2010/63/EU. The completed ARRIVE checklist is provided in Supplementary File S1.

### 2.2. Eligibility Criteria

#### 2.2.1. Inclusion Criteria

- Healthy white male BALB/c mice, 12 weeks old, weighing ~30 g.
- No clinical signs of systemic illness or infection;
- No cranial deformities or previous cranial surgery;
- Ability to tolerate anesthesia and surgical procedures without complications.

#### 2.2.2. Exclusion Criteria

- Pre-existing health issues, signs of infection, inflammation, or weight loss;
- Any intraoperative complications such as dural perforation or excessive bleeding;
- Failure to achieve proper wound closure or development of wound dehiscence;
- Signs of postoperative infection or abnormal healing;
- Inability to tolerate the PBM procedure under anesthesia.

### 2.3. Animal Model, Sample Size, and Randomization

Male BALB/c mice (12 weeks old, ~30 g) were selected due to their reliable immune profile, genetic uniformity, and relevance to human disease models [39,40], particularly in regeneration studies [16,28,40,41]. Their calvarial bones closely resemble the human jawbone, making them suitable for osteoregenerative research [42]. This model supports reproducibility while reducing biological variability, consistent with the Reduction principle of the 3Rs [43]. It is well validated for assessing PBM-induced bone healing [16,28,40,41], thereby enhancing translational relevance.

Male mice were used to avoid hormonal influences on bone formation, including estrogen-related periosteal inhibition in females [44,45]. White-skinned mice were chosen to reduce photonic energy absorption by pigmented skin [46]. Mice were 12 weeks old because BALB/c reach adulthood at this age, with closed calvarial sutures and mature bone hormone levels [47]. Aging, defined as six months or older, is associated with reduced bone density [48].

A total of 120 male BALB/c mice were included. Sample size was determined in consultation with a biostatistician, with 10 mice per each group as approved. All research data and materials were secured using encrypted, password-protected systems. Electronic data is stored exclusively on institution servers, and physical access is strictly restricted.

Animals were randomly assigned to groups using computer-generated block randomization to ensure equal group sizes and reduce selection bias [49]. Each group received a unique alphanumeric code. Outcome assessors and data analysts were blinded to allocation.

### 2.4. Animal Housing and Welfare

Prior to treatment, mice were group housed four per cage under standard laboratory conditions. These included a 12 h light/dark cycle controlled by an automatic timer, an ambient temperature of 22 ± 2 °C, and ventilation via an exhaust system. After surgery, mice were housed individually to prevent interference with healing and to allow accurate monitoring.

Animals had ad libitum access to water and a commercial diet throughout the study. All procedures were conducted in strict accordance with the “Guiding Principles for the Care and Use of Laboratory Animals.” The in vivo research complied with ethical principles, EU Directive 2010/63/EU, and relevant Italian animal welfare legislation. Procedures adhered to the 3Rs principles (Replacement, Reduction, and Refinement) and followed the ARRIVE 2.0 guidelines for transparent and reproducible reporting of in vivo experiments [50,51].

Animal welfare and dignity were respected at all times. Procedures aimed to minimize harm and maximize benefit for both scientific outcomes and animal well-being. Welfare was systematically monitored, including assessment of pain [52], distress, behavioral changes, locomotor activity, and water/food intake. In cases of unexpected mortality, autopsies were performed. Reserved mice (*n* = 12) were used to maintain group sizes and data integrity, consistent with the 3Rs principle.

### 2.5. Animal Anesthetic and Sedation Protocol

For surgical procedures, all mice were anesthetized via intraperitoneal injection of a ketamine and xylazine mixture (ketamine: 100 mg/kg body weight; xylazine: 10 mg/kg body weight) diluted in sterile saline. Each animal initially received 0.1 mL of the anesthetic solution. Additional doses of 0.01–0.02 mL were administered if signs of recovery were observed during the procedure.

For PBM irradiation treatments, which lasted 60 s per session, mice were sedated using isoflurane gas. Sedation was achieved in an induction chamber (Metrex Research, Romulus, MI, USA; volume: 725 mL). Isoflurane was delivered at a concentration of 5.00 ± 0.51%. Adequate sedation was typically reached after 35.70 ± 6.95 s at 87.6 kPa and 20 °C.

### 2.6. Animal Euthanasia Protocol

All animal procedures complied with the European Directive 2010/63/EU on the protection of animals used for scientific purposes. The study was approved by the institutional ethics committee in accordance with European Directive 2010/63/EU [53] and the Federation of European Laboratory Animal Science Associations (FELASA) [54].

Mice were euthanized via carbon dioxide inhalation in an approved chamber, using a flow rate of 2.0 L/min (~20% chamber volume/min) to minimize distress. Once unconsciousness was confirmed, the flow was increased to 10.0 L/min to ensure complete respiratory cessation [55].

Death was verified by absence of movement and respiration and was followed by a secondary physical method, either cervical dislocation or cardiac puncture, to confirm and ensure irreversibility. These procedures were performed in accordance with FELASA and AVMA guidelines [56].

### 2.7. Type of Biomaterial (Bone Graft) and Barrier Membrane

In this study, a sterile Geistlich Bio-Oss^®^ (0.25 g, small granules, particle size 0.25–1 mm) and a resorbable bilayer collagen membrane (Bio-Gide^®^) (13 mm × 25 mm) (Geistlich Pharma AG, Wolhusen, Switzerland) were used.

Bio-Oss^®^ is widely employed in guided bone regeneration (GBR) protocols and animal models of calvarial bone defects due to its high dimensional stability, slow resorption rate, and biocompatibility [57]. It was selected for this study because it demonstrates approximately 25% greater dimensional stability compared to autogenous bone in calvarial defect models, reducing variability in graft volume resorption and remodeling outcomes [58].

Additionally, Bio-Oss^®^ hydrophilic properties enable rapid and homogeneous hydration with blood or physiological fluids. This promotes early integration and provides uniform handling characteristics, which are crucial for experimental standardization [13,59].

The Bio-Gide^®^ membrane serves as a biocompatible, resorbable barrier that supports guided tissue regeneration by preventing soft tissue ingrowth and stabilizing the underlying graft material [60].

The combined use of Bio-Oss^®^ and Bio-Gide^®^ in the defect model allows controlled bone regeneration while minimizing confounding biological variability, facilitating the assessment of PBM effects.

### 2.8. Surgical Description for Calvaria Critical Bone Defect

Following anesthesia, hair over the calvarial region was shaved using an electric clipper. A semilunar incision was made in the anterior scalp, allowing elevation of a full-thickness flap in the posterior direction to expose the calvarial bone.

A CSD measuring 5 mm (0.5 cm) was created using a piezoelectric surgical device under continuous sterile saline irrigation. The defect area was first marked using a 5 mm biopsy punch to standardize both location and size [61]. Special care was taken to avoid injury to the dura mater and to preserve the sagittal suture. Soft tissue was repositioned and sutured without tension using 5/0 resorbable polyglycolic acid sutures.

PBM therapy was administered according to the experimental groups’ assignments described in Section 2.9. All surgical procedures and PBM applications were performed by the same clinician, experienced in both surgery and PBM therapy.

Postoperatively, animals were placed on a clean, pre-warmed heating pad to facilitate recovery from anesthesia. Mice were continuously monitored for two hours (h) for any signs of respiratory distress. Once fully responsive, they were returned to clean cages with free access to food and water.

### 2.9. Experimental and Controls Grouping

Animals were randomly allocated into 12 experimental groups according to the type of intervention and healing duration (30 or 60 days). A 980 nm PBM protocol was administered using either a Standard Gaussian (ST) or AB 2799 Flattop (FT) beam profile (Doctor Smile–LAMBDA Spa, Vicenza, Italy). The nominal laser power output was set at 0.6 W on the device interface. Irradiation was delivered for 60 s in a continuous emission mode, with three treatment sessions per week for two consecutive weeks [28]. The PBM treatment protocol was adapted from Hanna et al. 2019 [28]. Control groups received no laser irradiation (defect only and defect + Bio-Oss^®^). Animals were sacrificed at either 30 or 60 days post-treatment for histological and immunohistochemical analyses.

**Figure 1:**
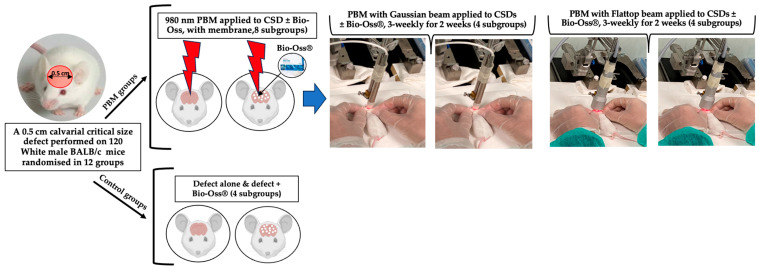
Schematic representation of the experimental design, including control groups (defect (CSD) alone and Bio-Oss^®^ alone) and interventional groups treated with PBM delivered with ST or FT handpieces, with or without Bio-Oss^®^. PBM was applied three times a week for two consecutive weeks. Created by the first author for illustrative purposes.

Table 1 and Figure 1 provide detailed information on the interventional groups and the PBM irradiation treatment protocol.

### 2.10. PBM Laser Device and Its Delivery Systems

The laser device employed in this study was the Wiser wireless diode laser (Doctor Smile–LAMBDA Spa, Vicenza, Italy), emitting photonic energy at a wavelength of 980 nm. This near-infrared (NIR) wavelength was selected due to its ability to penetrate biological tissues up to approximately 2.5 cm, making it particularly suitable for targeting deeper-seated tissues such as bone [62]. All equipment was purchased from Doctor Smile–LAMBDA Spa (Vicenza, Italy).

Two distinct handpieces, ST and FT, each producing different beam profiles, were used to irradiate CSD. The FT handpiece delivers a uniform energy distribution over a circular spot with a 1 cm diameter (0.785 cm^2^), providing homogeneous irradiance as specified by the manufacturer [28]. In contrast, the ST handpiece generates a Gaussian beam profile with centrally peaked intensity across the same 0.83 cm diameter spot (0.50 cm^2^). This Gaussian profile results in higher peak irradiance and fluence at the center, with a significant radial decrease in intensity.

The beam diameters were verified in contact mode (0 mm working distance) using a laser beam profiler (Gigahertz-Optik, model BGP-US83-SP32U, Teltow, Germany. The 2D spatial intensity distribution was recorded to confirm the FT beam profile. The mean area (cm^2^) was calculated from the measured diameter.

For standardized measurement purposes, a masking ring with a 0.5 cm diameter opening was placed between each laser handpiece and the power meter to record the actual energy striking the skin surface directly overlying the CSD.

Table 2 summarizes the laser device specifications, PBM dosimetry, and beam characteristics for both FT and ST handpieces used in this study. The therapeutic power was measured with a laser power meter (Gentec-EO TUNER, GentecElectro-Optic Inc., Quebec City, QC, Canada). Average irradiance was calculated by dividing the corrected power reaching the target surface by the beam area striking the CSD. Average fluence was obtained by multiplying the average irradiance by the irradiation time.

The data below point out that, while the laser was set to an identical output of 0.6 W in each case, apparent losses in the ST handpiece result in a measured output of only 0.42 W with this system. This makes the irradiation different, even though a clinician would believe that the same power was being delivered.

The laser device was calibrated at the start of each treatment session to ensure consistent energy delivery to the target tissue throughout the study.

### 2.11. A Novel Pilot Study for PBM-Laser Dosimetry Reaching Target Surface Area

A pilot ex vivo study was conducted to validate the PBM dosimetry protocol for delivering the therapeutic laser energy reaching the target (CSD) surface area. Four white mice were used to assess laser energy transmission to calvarial defects under two conditions. In the first condition (*n* = 2), a 5 mm calvarial defect was created and left unfilled, followed by repositioning and suturing of the overlying shaved skin (thickness approximately 2 mm, measured with a caliper). In the second condition (*n* = 2), the defect was filled with approximately 2 mm of Bio-Oss^®^ granules (particle size: 0.25–1 mm) [63,64] and covered with a 0.4 mm Bio-Gide^®^ membrane [64], followed by repositioning and suturing of the overlying shaved skin. For each mouse, multiple measurements of laser power transmitted through the overlying layers were recorded. The mean ± standard deviation (SD) of these repeated readings was calculated to assess the precision of the PBM dosimetry protocol.

**Figure 2:**
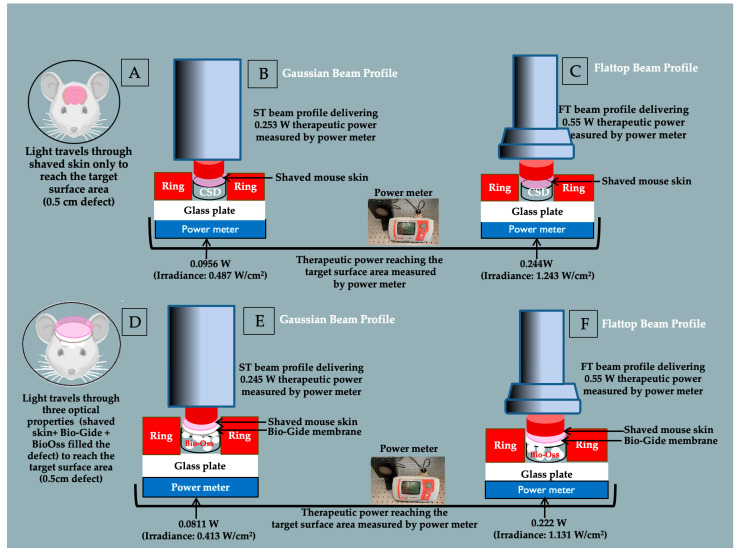
Schematic illustration of a novel pilot ex vivo study for standardized photobiomodulation (PBM) dosimetry reaching the target surface under two experimental conditions related to the main study. Created by the first and second authors for illustrative purposes. **Condition one** (**A**): Laser light pathway reaching the 0.5 cm defect surface area through shaved skin. Panels (**B**,**C**) show measurements using two different beam profiles: (**B**): Gaussian beam profile (ST) delivering 0.253 W therapeutic power measured power reaching the target: 0.0956 W (irradiance: 0.487 W/cm^2^); (**C**): Flattop beam profile (FT) delivering 0.55 W therapeutic power; measured power reaching the target: 0.244 W (irradiance: 1.243 W/cm^2^). **Condition two** (**D**): Laser light pathway reaching the 0.5 cm defect surface area through shaved skin, Bio-Gide^®^ membrane, and Bio-Gide^®^ -filled defect. Panels (**E**,**F**) show measurements using two different beam profiles: (**E**): Gaussian beam profile (ST) delivering 0.253 W therapeutic power measured power reaching the target: 0.0811 W (irradiance: 0.413 W/cm^2^); (**F**): Flattop beam profile (FT) delivering 0.55 W therapeutic power measured power reaching the target: 0.222 W (irradiance: 1.131 W/cm^2^). **Note: Therapeutic power output values include approximately 10% transmission loss through the supporting glass plate**.

Following the euthanasia of the four mice, the area of interest (0.5 cm) was placed on a glass plate and isolated with a ring of 0.5 cm. A 980 nm at nominal power output of 0.55 W for FT and 0.253 W for ST measured by power meter (taking into account the 10% glass absorption) applied in CW directly in contact with sutured mouse shaved skin with and without biomaterials using both ST and FT beam profiles. The laser power transmitted through the overlying layers to the bottom of the defect was measured directly using a calibrated optical power meter (Gentec-EO TUNER, GentecElectro-Optic Inc, Quebec City, QC, Canada). A schematic illustration of the experimental setup for ex vivo PBM irradiation and laser power measurement is shown in Figure 2. These measurements allowed accurate quantification and comparison of energy transmission between mouse shaved skin-only and combined shaved skin, Bio-Oss^®^ and Bio-Gide^®^ membrane conditions.

Infrared thermography was performed using a calibrated FLIR E4 IR camera (FLIR Systems, INC., FLIR ONE Pro-iOS, Portland, OR, USA) at 1 m distance in a temperature-controlled room (22–24 °C) to monitor thermal changes on mouse skin during irradiation.

The ex vivo validation model measured the actual therapeutic power reaching the base of the calvarial defect under two conditions: shaved mouse skin alone and shaved mouse skin with Bio-Gide^®^ membrane and Bio-Oss^®^ filling the defect, using ST and FT beam profiles (Figure 2). These data confirmed the actual therapeutic power output at the defect surface area and guided the irradiation parameters consistently applied in all in vivo experimental groups. Beam profile and the optical properties of the overlying layers were the only variables affecting power transmission, ensuring controlled and reproducible dosimetry throughout the study.

### 2.12. Outcome Measures

#### 2.12.1. Primary Outcome

To assess the efficacy and validity of a novel 980 nm PBM dosimetry protocol in delivering precise, reproducible energy to the target tissue surface area in vivo, as determined by dosimetry validation and associated osteogenic response.

#### 2.12.2. Secondary Outcomes

- Histological analysis: assessment of new bone formation through qualitative evaluation of tissue structure and organization, and quantitative measurement of the area of new bone.
- Immunohistochemical (IHC) markers:
  ○ CD34: evaluated as an indicator of vascular presence and neovascularisation, quantified by counting CD34-positive vessels per field.
  ○ Gli1: marker of early osteoprogenitor and skeletal stem cell activation; quantified number of Gli1-positive cells per field.
  ○ Runx2: evaluated for early- to mid-stage osteoblast differentiation by IHC without quantification
  ○ OCN: marker of late-stage osteoblast maturation and matrix mineralization; quantified by the number of OCN-positive cells per field.
  ○ CTSK: marker of osteoclast activity in bone remodeling; quantified by the number of CTSK-positive osteoclast per field.
- Comparative effect of FT vs. ST beam profiles with or without Bio-Oss^®^ grafting on bone regeneration.
- Influence of PBM on the Bio-Oss^®^ grafting, whether there is any synergistic effect to enhance bone regeneration

### 2.13. Assessment Tools

#### 2.13.1. Histological Analysis

Histological sections stained with hematoxylins and eosin (H&E) were digitized using a Leica DFC 320 microscope (Leica Microsystems, Wetzlar, Germany) at 10× magnification. For each experimental group, six random fields covering the region of interest (ROI), the 0.5 cm diameter calvarial defect, were captured. Using ImageJ software, version 1.5c (NIH, Bethesda, MD, USA), the area occupied by newly formed bone tissue in each image was measured.

The total defect area within each image was also measured, and the percentage of new bone formation was calculated. This percentage quantifies the extent of bone regeneration within the defect [65]. For each group, measurements from 10 images obtained from 10 different animals were averaged to determine the mean bone regeneration and its variability [66,67].

After euthanasia at 30 and 60 days, skulls were collected and fixed in 10% neutral buffered formalin diluted in phosphate-buffered saline (PBS) for 72 h. To enhance fixative perfusion, the mandible was disarticulated from the maxilla, and formalin was injected using an insulin syringe (31-gauge needle) through the orbital cavity, the foramen magnum, and a 2 mm hole created at the level of the palatine bone near the sella turcica.

Samples were then washed in PBS for 48 h with buffer changes every 4 h. Subsequently, specimens were decalcified in a solution of 37% hydrochloric acid and 98% formic acid for 3 days, with solution renewal every 24 h. This acid-based decalcification protocol balances efficient mineral removal and preservation of tissue morphology [68,69].

Skulls were embedded in paraffin, and 5 μm coronal sections were cut using a rotary microtome (Reichert Jung Leica 2040, Leica Biosystems, Nussloch, Germany). Sections were stained with hematoxylin and eosin (H&E) for general morphological evaluation and Gomori’s Trichrome (GT) staining to visualize collagen fibers and extracellular matrix organization. GT is especially useful for distinguishing mineralized bone from connective tissue, providing structural context for bone remodeling [70,71]. The slides were observed and analyzed under a Leica DFC 320 light microscope.

#### 2.13.2. Immunohistochemistry of Bone Molecular Biomarkers

To assess collagen type, cell populations, and bone healing in normal and damaged areas, serial sections were processed using specific polyclonal and monoclonal antibodies: rabbit pAb anti-osteocalcin (Abcam plc, Cambridge, MA, USA, ab93876, 1:200) [72], recombinant rabbit mAb anti-cathepsin K (Abcam, USA [EPR24829-101]; ab300569, 1:1000) [73], recombinant rabbit mAb anti-Gli1+ (Abcam plc, Cambridge, MA, USA [HL247]; ab289368, 1:300) [74], rabbit pAb anti-collagen III (Chemicon, Temecula, CA, USA) [75], and mouse mAb anti-CD34 (Zymed Inc., San Francisco, CA, USA; Clone BL-35C) [72]. Negative controls were prepared by substituting the primary antibody with TBS or nonimmune sera.

Bone defect specimens were collected at 30 and 60 days post-treatment. After euthanasia, calvarial blocks were rinsed in PBS, fixed in 10% buffered formalin for 24 h, decalcified in 10% EDTA (pH 7.4) at 4 °C for 3–4 weeks, and processed through ethanol dehydration, xylene clearing, and paraffin embedding. Serial 5 µm sections were mounted on silanized SuperFrost^®^ Plus slides (Thermo Fisher Scientific, Waltham, MA, USA) and stored until staining.

For immunohistochemistry, sections were dewaxed, rehydrated, and subjected to antigen retrieval in EDTA buffer (pH 9.0) using microwave heating. After cooling, blocking buffer (0.3% Triton X-100, 0.5% BSA in PBS) was applied for 1 h, followed by overnight incubation with primary antibodies in TBS with 0.1% BSA. After PBS washes, antibody binding was detected with ABC-peroxidase, biotin-conjugated secondary antibodies (1:200), streptavidin-HRP, and DAB, followed by hematoxylin counterstaining. Negative controls omitted the primary antibody.

For Runx2 staining, dewaxed and rehydrated sections underwent citrate buffer retrieval (10 mM, pH 6.0) at 95 °C for 20 min. Slides were blocked (0.3% Triton X-100, 0.5% BSA) and incubated overnight with rabbit anti-Runx2 (Santa Cruz Biotechnology, Dallas, TX, USA; sc-390351; 1:50) [75], followed by goat anti-rabbit Alexa Fluor 594 (Invitrogen, Carlsbad, CA, USA; A11037; 1:500) [74]. Images were obtained with a C2 Plus confocal microscope, and Runx2 fluorescence intensity was quantified in five high-power fields (400×) using ImageJ (mean ± SD).

For quantification, fluorescence (Runx2) and DAB-stained sections (OCN, CTSK, Gli1, CD34) were further processed in ImageJ (NIH, Bethesda, MD, USA). Images were converted to 8-bit grayscale, and the background was subtracted using a rolling ball radius of 50 pixels. For Runx2, a manual threshold was defined using negative control sections and applied consistently across all images in the batch to include clearly positive nuclei while excluding background. For DAB-stained markers, thresholding was performed using the Yen algorithm and applied consistently across all images. Thresholded images were converted to binary, and the “Analyze” menu followed by the “Measure” function in ImageJ was used to calculate the percentage of positive area. For particle-based counts (e.g., CD34+ cells), particles smaller than 10 μm^2^ were excluded. Quantification was performed blinded to the experimental group, and the results were reported as mean ± SD.

Immunohistochemistry evaluated Runx2, OCN, CTSK, Gli1, and CD34 expression. CD34+ mononuclear cells were assessed in perivascular regions. Marker expression was evaluated qualitatively at 40× magnification and quantitatively by counting positive cells in five representative high-power fields (400×) per defect region. For OCN, CTSK, Gli1+, and CD34, counts were performed in reactive-regenerative areas using a light microscope (Carl Zeiss, Jena, Germany) with a 40× objective and 10× eyepiece containing a 10 × 10 square graticule (62,500 μm^2^ per square). Ten fields were analyzed per compartment, excluding margin cells. Results were expressed as IHC-positive cells per 62,500 μm^2^ and area percentage of positive staining (ImageJ), reported as mean ± SD.

### 2.14. Statistical Analysis

All quantitative data are presented as mean ± standard deviation (SD). The study included 120 mice allocated into 12 independent groups (10 mice per group). Each treatment condition was divided into two subgroups, with mice euthanized at either 30 or 60 days. Each mouse contributed the mean of five measurements as a single independent data point.

To analyze differences across treatment groups and time points, a two-way ANOVA (between-subjects) was used to assess the main effects of treatment, time, and their interaction. When a significant interaction was detected (*p* ≤ 0.05), post-hoc comparisons were performed using the Holm–Šídák multiple-comparison test.

Histological and immunohistochemical scores, which are ordinal variables, were compared between groups using the Mann–Whitney U test (*p* ≤ 0.05).

For the antibody-expression markers (Runx2, OCN, CTSK, Gli1, and CD34), data normality was assessed using the Shapiro–Wilk test, followed by one-way ANOVA at each time point and Holm–Šídák post-hoc tests (* *p* < 0.05; ** *p* < 0.01; *** *p* < 0.001; **** *p* < 0.0001).

All statistical analyses were performed using GraphPad Prism 9 for macOS (version 9.3.1; GraphPad Software Inc., San Diego, CA, USA).

## 3. Results

### 3.1. Pilot Study Results: Therapeutic Power Reaching Target Surface Area

Therapeutic power output was measured using a 0.5 cm ring to isolate the area of interest, with a supporting glass plate that absorbed approximately 10% of the power. After correcting for glass absorption, the actual delivered power was approximately 0.56 W for the FT and 0.256 W for the ST. All power output values were measured using a calibrated power meter.

Table 3 presents the results of the pilot investigation, detailing the PBM dosimetry reaching the CDS surface area. Due to differences in beam profile and optical attenuation through shaved mouse skin and biomaterials, the therapeutic power reaching the target surface area differed between the FT and ST, despite identical irradiation parameters.

For each mouse, multiple measurements of transmitted laser power were performed. All repeated readings were identical (SD = 0 within the resolution of the power meter), indicating no detectable measurement variability under the experimental conditions and excellent repeatability of the PBM dosimetry protocol in this pilot sample.

Although the ST and FT beams had different nominal beam diameters and total beam areas (0.50 cm^2^ and 0.785 cm^2^, respectively), the effective irradiated area was standardized by the use of a 0.5 cm diameter ring defining the CSD. Consequently, the area receiving irradiation was identical for both beam profiles and corresponded to the defect area (0.196 cm^2^). All irradiation parameters were therefore normalized to this effective irradiated area.

**Figure 3:**
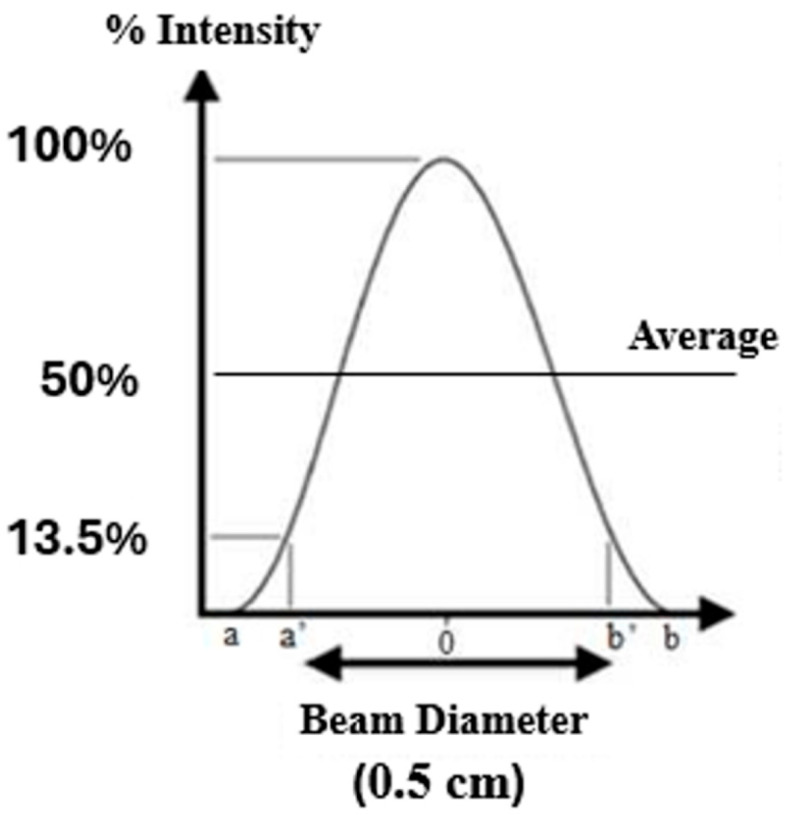
Schematic illustration of a Gaussian distribution. The central beam has an intensity of twice the measured average. On the other hand, the periphery of the beam has 13.5% of the peak intensity. This disparity will produce a vastly different PBM effect on target tissues. The image is the authors’ production. Created by the first and second authors for illustrative purposes.

Average irradiance and fluence were calculated from the therapeutic power reaching the target area divided by the standardized beam area. A Gaussian beam (ST), by definition, has a peak power in the center of exactly twice the average and 13.5% of peak power at the periphery [76,77,78,79] (Figure 3). By contrast, the peak and average powers are the same for a flattop beam. While not measured directly, these values were used in Table 3 to represent theoretical peak values

It is apparent from these data that the overlying mouse skin diminishes the effective energy reaching the target tissue (CSD) by more than 50%. This is in line with most previous studies and highlights the need for careful measurement and calculation of administered dosage [80,81].

To ensure that PBM effects were not confounded by thermal changes, infrared thermography was employed to monitor surface temperature at the defect site during the 60 s irradiation period. Ambient room temperature was maintained at 22–24 °C. Skin surface temperature at the defect site remained stable, fluctuating between 25 and 28 °C, with no measurable increase attributable to laser exposure.

### 3.2. Histological Assessment of Bone Regeneration

To evaluate the effects of laser therapy on bone regeneration, we conducted histological analyses on 12 experimental groups (Table 1). The histological evaluation was performed using a Leica DFC 320 microscope at ×10 magnification, with six random fields (ROI) per each group analyzed.

#### 3.2.1. H&E Staining

**Figure 4:**
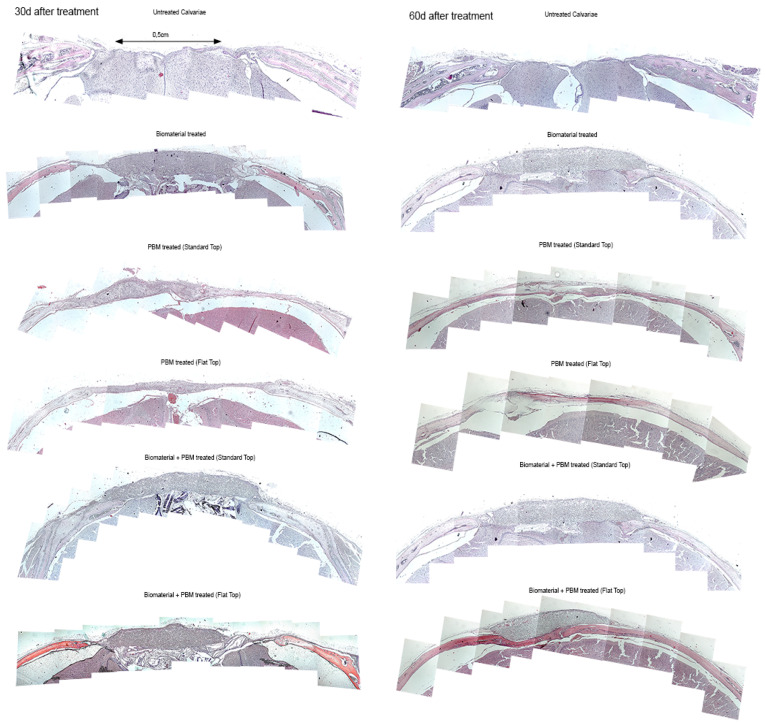
Histological evaluation of calvarial bone regeneration by Hematoxylin & Eosin staining at 30- and 60-days post-PBM treatment. Untreated (defect only), Bio-Oss^®^ alone, PBM-ST, PBM-FT, PBM-ST + Bio-Oss^®^, and PBM-FT + Bio-Oss^®^ groups. Nuclei appear Blue/purple (hematoxylin), and cytoplasm, collage, and extracellular matrix appear pink/red (eosin). Representative panoramic sagittal sections of mouse calvaria from different treatment groups are shown. Images highlight newly formed bone and tissue morphology. Sections were captured at 10× objective (field of view ~1.5 mm). Scale bar = 200 µm.

Histological evaluation of calvarial sections revealed distinct regenerative patterns among the experimental groups, particularly between the PBM-FT and PBM-ST modalities at 30 and 60 days post-treatment (Figure 4).

The untreated control consistently shows a wide tissue defect, persistent inflammation, and poor collagen organization, indicating minimal natural healing. The Bio-Oss^®^-only group exhibits moderate improvement, with partial defect closure and early scaffold integration at 30 days, progressing to connective tissue formation and partial remodeling by 60 days; however, complete re-epithelialization remains limited.

In contrast, the PBM-treated groups demonstrate markedly improved healing responses. The PBM-ST treatment results in increased granulation tissue formation, greater fibroblast activity, and emerging vascular structures by 30 days, with more organized collagen and reduced inflammation by 60 days. However, the PBM-FT variant produces more uniform and mature tissue regeneration, characterized by denser collagen deposition, fewer inflammatory cells, and a more continuous epithelial layer at both time points. The FT beam likely ensures more homogeneous light distribution and deeper tissue stimulation, which enhances fibroblast proliferation and collagen remodeling compared to the standard setup.

When PBM is combined with the biomaterial, the synergistic effect is most evident. At 30 days, both Bio-Oss^®^ + PBM groups show substantial defect bridging and tissue organization, but the FT combination displays more advanced collagen maturation and less inflammatory infiltration. By 60 days, the Bio-Oss^®^ + PBM-FT group exhibits the most complete and mature repair, near-normal tissue architecture, dense and well-aligned collagen bundles, and fully re-epithelialized surfaces, surpassing all other groups.

In summary, while both PBM modalities enhance healing compared to untreated or Bio-Oss^®^-only treatments, the PBM delivered with FT, particularly when combined with the Bio-Oss^®^, yields superior and more consistent histological outcomes, suggesting more effective tissue regeneration and remodeling over time.

#### 3.2.2. Masson Trichrome (MT) Staining

The MT staining is a commonly used method in bone histology and allows tissue identification by different coloring as well as by morphological identification. In context, the light blue/blue color indicates the regenerated bone (early bone tissue), collagen fibers, or osteoid, while the red-colored micro-areas designate the mature bone.

**Figure 5:**
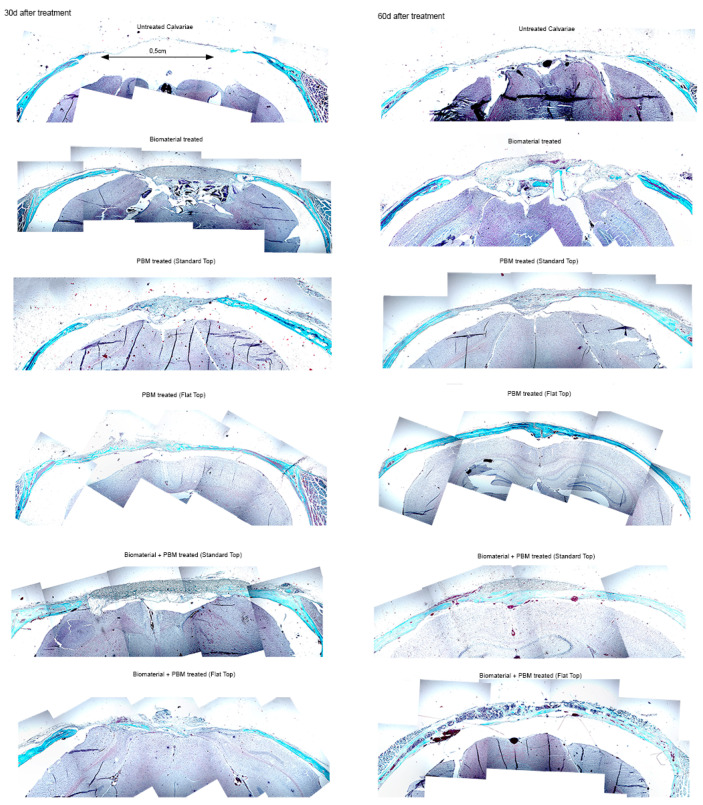
Representative Masson’s Trichrome stained histological sections of calvarial defects at 30 and 60 days post-treatment. Images show comparative bone regeneration among groups: Untreated (defect only), Bio-Oss^®^ alone, PBM-ST, PBM-FT, PBM-ST + Bio-Oss^®^, and PBM-FT + Bio-Oss^®^ groups. Collagen and fibrous connective tissue are stained blue, osteoid and new bone matrix red, and nuclei dark purple. Images were captured at 10× objective (field of view ≈ 1.5 mm)**.** Scale bar = 200 µm.

The results of the MT staining revealed distinct qualitative differences in bone regeneration, collagen deposition, and tissue organization among the experimental groups at 30 and 60 days after treatment. It provides further confirmation of different regenerative responses among the experimental groups (Figure 5). These results were comparable to those observed after H&E staining.

In the untreated calvarial bone defects, minimal regenerative activity (<10%) was evident at both time points, with the defect space primarily filled by loose connective tissue and spare blue-stained collagen fibers, indicating poor intrinsic healing capacity. In contrast, the Bio-Oss^®^-treated specimens exhibited partial defect filling at 30 days, primarily with early fibrous tissue formation and limited osteoid deposition (approximately 20–30%), surrounding residual biomaterial particles. After 60 days, the defect area showed more intense blue staining and localized matrix organization, indicating progressive collagen deposition and early osteoid development, but incomplete repair.

In PBM-treated groups (via ST and FT handpieces), an enhanced tissue osteogenic response was observed compared with untreated controls. After 30 days, the PBM-ST group showed evidence of increased red-stained osteoid tissue deposition at the defect margins (approximately 40–50% of the defect area) and elevated cellular activity. In contrast, the PBM-FT group exhibited a more uniform distribution and continuous newly formed tissue across the defect, with denser collagen accumulation and improved matrix continuity. At 60 days, both PBM-ST and FT groups demonstrated a substantial increase in new bone-like matrix (>60%). Notably, the PBM-FT group exhibited mature tissue characteristics, including a well-organized collagen network and areas of mineralized bone matrix, indicating that PBM alone effectively stimulated bone regeneration.

The combination of Bio-Oss^®^ and PBM-treated groups displayed the most pronounced regenerative outcomes. At 30 days, both PBM-ST and FT configurations demonstrated extensive active tissue ingrowth within and around the Bio-Oss^®^ matrix, with prominent red and blue staining corresponding to concurrent collagen synthesis and osteoid formation in approximately 60–70% of the defect area. At 60 days, these defects exhibited nearly complete bone bridging and defect closure (>80%), with dense and organized collagen fibers, advanced mineralization, and mature bone tissue with minimal residual defect space. Notably, the Bio-Oss^®^ + PBM-FT group exhibited the highest degree of mature bone-like tissue architecture, with superior integration and continuity with the surrounding native bone, indicating a synergistic effect between PBM photonic energy (biostimulation) and the Bio-Oss^®^ scaffold in enhancing bone regeneration.

Overall, PBM therapy positively influenced the transition from early fibrous tissue deposition to mature bone formation, leading to accelerated calvarial defect healing over time.

### 3.3. Immunohistochemistry Analysis of Molecular Biomarkers

To complement the histological analysis and further validate our findings, we performed immunohistochemistry staining for several key molecular biomarkers involved in bone regeneration: GLi1, CD34, Runx2, OCN, and CTSK. These markers were selected to assess various stages of osteogenesis and bone remodeling.

The results from these molecular markers were consistent with our histological findings, confirming that PBM-FT treatment combined with Bio-Oss^®^, in particular, significantly enhanced osteogenesis and matrix remodeling, leading to more robust bone formation in the defect areas. Detailed results for each biomarker are outlined below.

#### 3.3.1. CD34- Stem/Progenitor Cell Marker

**Figure 6:**
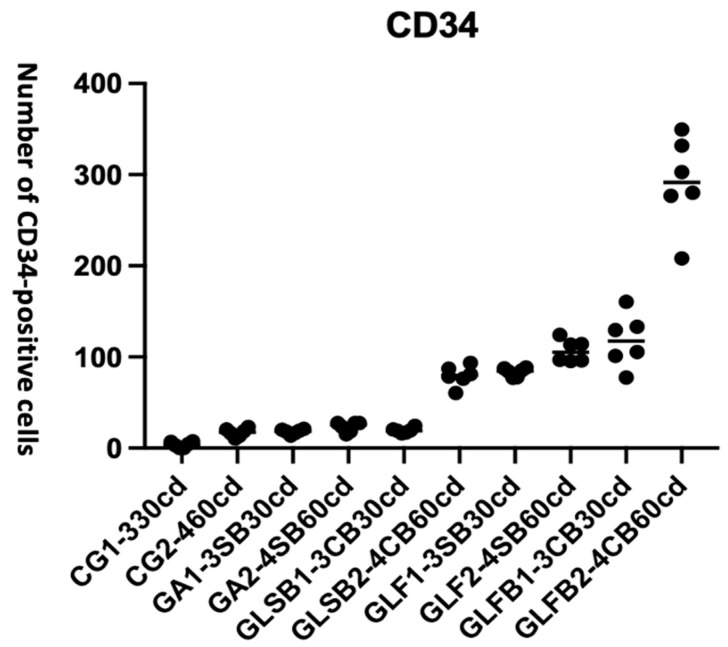
ANVOA results showing the number of CD34-positive cells across the experimental groups. The x-axis represents the interventional groups at the indicated timepoints, and the y-axis shows the number of CD34-positive cells (0–400 in intervals of 100). PBM-FT exhibited the highest number of CD34-positive cells compared to the other groups.

ANOVA results for CD34, the number of positive cells, resulted in significantly different between groups (F = 120.9, **** *p* < 0.0001) (Figure 6).

Table 4 presents the details of the quantitative data of group comparisons at 30 and 60 days.

PBM-FT combined with Bio-Oss^®^ significantly increased the number of CD34-positive cells at 30 days compared with PBM-FT without Bio-Oss^®^ at 60 days.

Moreover, PBM-FT showed superior performance in terms of CD34-positive cells at both 30 and 60 days compared with all other groups (**** *p* < 0.0001).

As early as 30 days, PBM-FT without Bio-Oss^®^ performed significantly better than PBM-ST without Bio-Oss^®^ (**** *p* < 0.0001) and PBM-ST with Bio-Oss^®^ (**** *p* < 0.0001). These differences were statistically significant.

From 30 to 60 days, no significant increase in CD34 positive cells was observed in any group. Within the PBM-FT groups, both with and without Bio-Oss^®^, the number of CD34-positive cells did not increase further, as levels were already high at 30 days. The CD34 values observed in PBM-FT groups (with and without Bio-Oss^®^) at 30 days were significantly higher than those measured in the GA group (**** *p* < 0.0001) and in the PBM-ST groups (with and without Bio-Oss^®^) at 60 days (*p* = 0.0178).

At 60 days, PBM-FT with Bio-Oss^®^ demonstrated the best overall performance compared with PBM-FT without Bio-Oss^®^ (**** *p* < 0.0001). The greatest statistically significant difference was observed for PBM-FT when compared with all other groups (*p* < 0.0001).

#### 3.3.2. GLi 1 Expression

**Figure 7:**
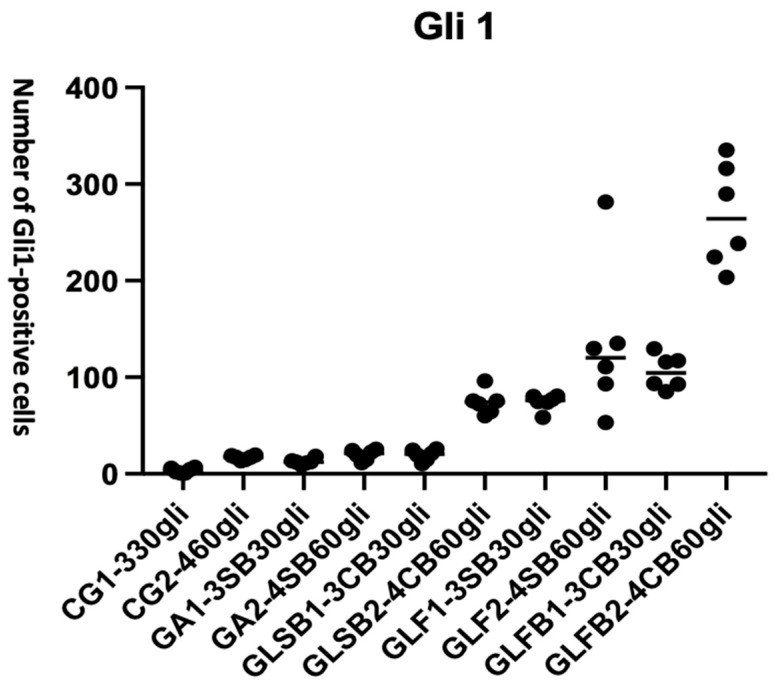
ANOVA analysis of Gli1-positive cell counts across the experimental groups. The x-axis represents the intervention groups at 30 and 60 days, and the y-axis indicates the number of Gli1-positive cells (0–400, intervals of 100). The PBM-FT + Bio-Oss^®^ group showed the highest number of Gli1-positive cells at both 30 and 60 days, with significantly greater expression compared to all other groups.

Gli1 is a transcription factor involved in Hedgehog signaling and is commonly expressed in early progenitor cells. ANOVA analysis showed a significant difference in the number of Gli1-positive cells among the groups (F = 41.86, **** *p* < 0.0001). The results are presented in Figure 7.

Table 5 outlines the details of the qualitative data of group comparisons at 30 and 60 days timepoints.

PBM-FT combined with Bio-Oss^®^ significantly increased the number of Gli1-positive cells at both 30 and 60 days compared with all other groups, indicating both early and sustained activation of tissue-healing pathways. These findings suggest that PBM-FT with Bio-Oss^®^ enhances Hedgehog signaling activity, potentially contributing to accelerated or improved bone regeneration.

Between 30 and 60 days, a significant increase in Gli1-positive cells was observed only in the PBM-FT groups, both with (**** *p* < 0.0001) and without Bio-Oss^®^ (*p* = 0.0338).

At 30 days, the presence of Bio-Oss^®^ enhanced the number of Gli1-positive cells in the PBM-FT group compared with the PBM-ST at 60 days (*p* = 0.0004).

Already at 30 days, the PBM-FT group without Bio-Oss^®^ showed significantly better results than both the ST with Bio-Oss^®^ (*p* = 0.0338) and the PBM-ST without Bio-Oss^®^ (**** *p* < 0.0001). Moreover, the performance of the PBM-FT, in terms of the number of antibody-positive cells, further increased at 60 days (**** *p* < 0.0001).

At 60 days, the PBM-FT with Bio-Oss^®^ achieved the best performance, even when compared with the same laser configuration without Bio-Oss^®^ (**** *p* < 0.0001). The most significant difference was observed for the PBM-FT group compared with all other experimental groups (**** *p* < 0.0001).

#### 3.3.3. Runx2 Expression

**Figure 8:**
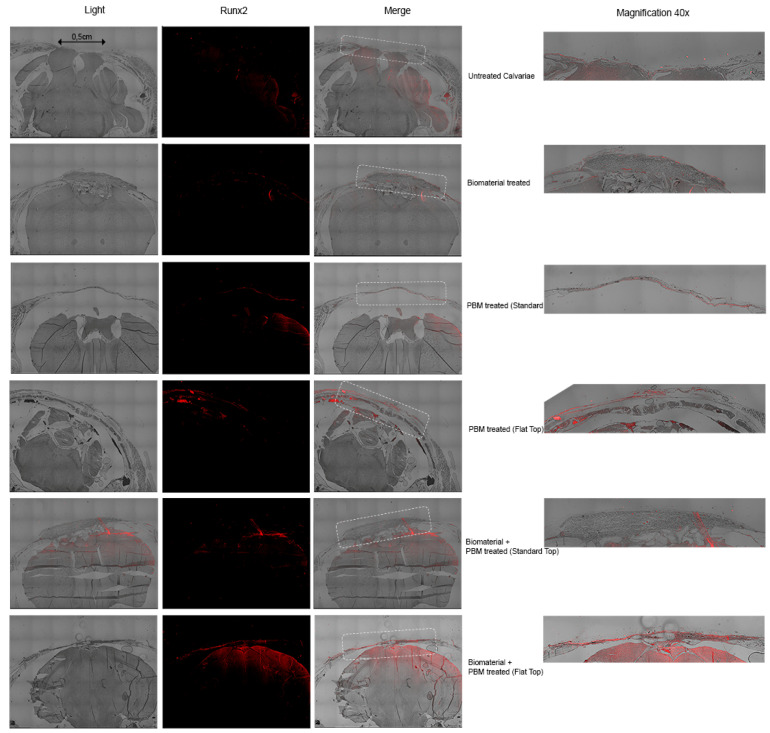
Immunofluorescence detection of Runx2 expression in calvarial defects at 30 days post-treatment. Representative images showing Runx2 immunostaining (red) in different treatment groups. Columns display light microscopy, Runx2 fluorescence, and merged images. Higher-magnification panels (40× objective) highlight regions of interest (ROIs, indicated by white boxes in 10× overview) to show detailed cellular expression patterns. Scale bars: 10× panels = 200 µm; 40× panels = 50 µm.

The immunofluorescent staining for Runx2 was performed to evaluate early osteogenic activity within the calvarial defect areas at 30 and 60 days after treatment. Runx2 expression, indicated by red fluorescence, revealed distinct variations among the experimental groups. All these data for 30 days are shown in Figure 8.

At 30 days, in the untreated calvarial defects, minimal or absent Runx2 labeling was observed, suggesting negligible osteogenic activation and limited recruitment of osteoprogenitor cells. The Bio-Oss^®^-treated specimens displayed weak but localized Runx2 fluorescence, primarily surrounding the implanted material, indicating an initial cellular response and early osteoblastic differentiation.

PBM-treated groups displayed markedly higher Runx2 expression, particularly at the defect margins and extending into the central region. The PBM-ST configuration demonstrated moderate fluorescence intensity, whereas PBM-FT showed more homogeneous and widespread Runx2 distribution throughout the defect area, reflecting a more uniform stimulation of osteogenic precursors.

The combined Bio-Oss^®^ + PBM-treated groups exhibited the strongest and most extensive Runx2 fluorescence signals. In the Bio-Oss^®^ + PBM-ST group, Runx2 expression was concentrated around the biomaterial particles, indicating enhanced osteogenic differentiation within the scaffold. The Bio-Oss^®^ + PBM-FT group showed the most intense and uniformly distributed fluorescence, covering nearly the entire defect area. This pattern suggests a synergistic interaction between PBM delivered with the FT beam profile and the Bio-Oss^®^ scaffold, resulting in pronounced activation of Runx2-mediated transcription and early osteoblast differentiation.

These findings confirm the synergetic effects of PBM therapy, particularly with the FT beam profile, with Bio-Oss^®^ enhancing early osteogenic response, and its combination with a Bio-Oss^®^ scaffold further amplifies Runx2 expression, accelerating the bone regeneration process during the initial healing phase.

**Figure 9:**
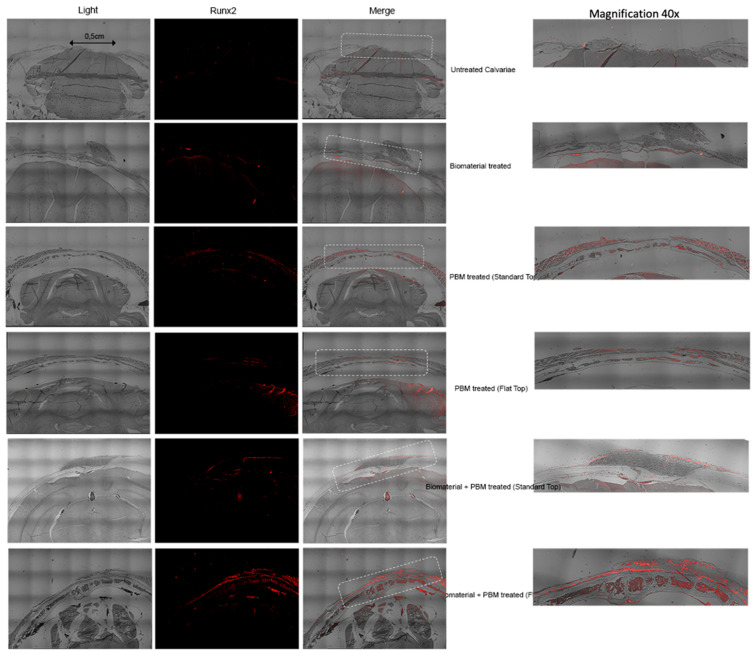
Immunofluorescence detection of Runx2 expression in calvarial defects at 60 days post-treatment. Representative images showing Runx2 immunostaining (red) across treatment groups: untreated defect, Bio-Oss^®^ treated, PBM-ST, PBM-FT, Bio-Oss^®^ + PBM-ST, and Bio-Oss^®^ + PBM-FT. Columns display bright-field images, Runx2 fluorescence, merged overlays, and 40× magnified views of representative regions of interest (ROIs, indicated by white boxes in the 10× overview panels). Scale bar: 10 x panels = 200 µm; 40× panels = 50 µm.

Figure 9 shows all the data for immunofluorescence detection of Runx2 expression in calvarial defects at 60 days post-treatment.

At 60 days after treatment, immunofluorescent labeling for Runx2 continued to demonstrate distinct differences among the experimental groups, reflecting the varying stages of bone regeneration and osteoblastic activity. In the untreated calvarial defects, Runx2 expression remained nearly absent, consistent with the persistence of unhealed fibrous tissue and minimal osteogenic signaling (Figure 9).

In the Bio-Oss^®^-treated specimens, weak and scattered Runx2 immunoreactivity was observed around residual biomaterial particles, suggesting delayed osteoblastic differentiation and incomplete scaffold-bone integration.

In contrast, the PBM-treated groups maintained moderate Runx2 fluorescence, although the signal appeared more localized compared with the 30-day stage, suggesting progression from early osteogenic activation to a more mature bone remodeling phase.

The PBM-ST group displayed discrete Runx2-positive regions along the newly formed bone edges, reflecting ongoing osteoblastic differentiation and matrix maturation; however, the PBM-FT group exhibited a broader distribution of the Runx2 signal across the defect interface, with stronger intensity in areas corresponding to active remodeling and mineralized tissue formation, confirming that PBM continues to enhance osteogenic activity during later healing stages.

The combined Bio-Oss^®^ + PBM-treated groups showed the most organized and mature tissue architecture accompanied by strong, localized Runx2 expression. In the Bio-Oss^®^ + PBM-ST group, Runx2 fluorescence was detected mainly around the margins of the newly formed bone and within the residual Bio-Oss^®^ matrix, indicating dynamic remodeling and integration processes.

Interestingly, the Bio-Oss^®^ + PBM-FT group presented the highest and most uniform Runx2 signal intensity within the regenerated bone region, highlighting sustained osteogenic stimulation and active remodeling at advanced healing stages. These findings suggest that PBM, particularly in combination with the Bio-Oss^®^ scaffold, not only accelerates early osteogenic differentiation but also maintains transcriptional activity associated with bone maturation and remodeling up to 60 days after treatment.

Runx2 expression was evaluated qualitatively by immunofluorescence; quantitative positive cell counts were not performed. Nevertheless, trends in osteogenic activity are supported by the quantitative analyses of CD34, Gli1, OCN, and CTSK.

#### 3.3.4. OCN Expression

**Figure 10:**
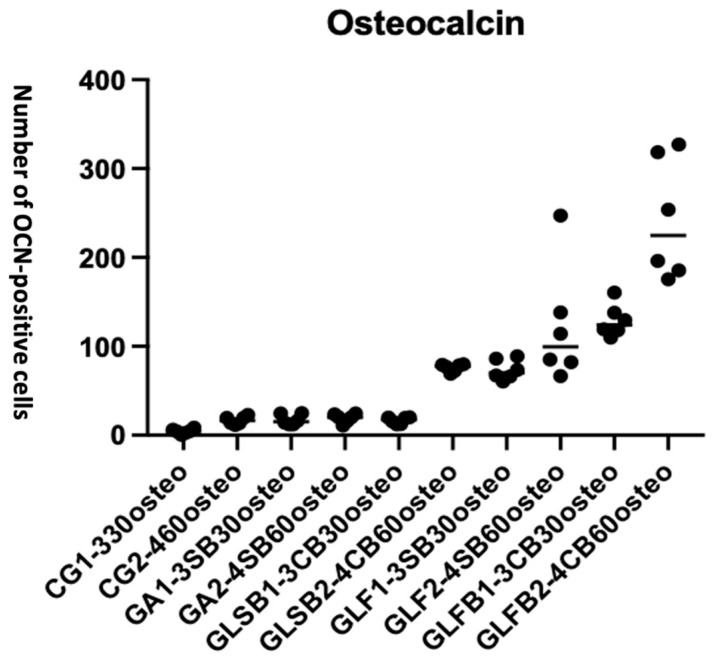
ANOVA analysis of OCN-positive cell counts (Quantification) across the experimental groups. The x-axis represents the intervention groups at 30 and 60 days, and the y-axis shows the number of OCN-positive cells (0–400, in intervals of 100). PBM-FT combined with Bio-Oss^®^ exhibited the highest OCN expression compared to other groups, indicating enhanced osteogenic differentiation.

ANOVA followed by multiple comparisons demonstrated a significant difference in the number of OCN-positive cells among the experimental groups (F = 35.76, **** *p* < 0.0001) (Figure 10).

Table 6 outlines the detailed quantitative comparisons between groups at 30 and 60 days timepoints.

OCN expression was lowest in the control and non-irradiated groups, while PBM-FT combined with Bio-Oss^®^ produced the highest number of OCN-positive cells. This increase reflects enhanced osteogenic differentiation in the PBM-FT + Bio-Oss^®^ group, supporting its superior capacity to promote bone-forming activity compared with all other treatments.

PBM-FT with Bio-Oss^®^ significantly increased the number of osteocalcin-positive cells at both 30 and 60 days. Notably, PBM-FT without Bio-Oss^®^ also produced higher osteocalcin expression than PBM-ST with Bio-Oss^®^ (*p* = 0.0495), and this effect became even more pronounced at 60 days (**** *p* < 0.0001).

In addition, the presence of Bio-Oss^®^ further enhanced OCN expression within the PBM-treated groups at 60 days, with a statistically significant increase observed in the PBM-FT + Bio-Oss^®^ group (**** *p* < 0.0001) compared with the PBM-ST + Bio-Oss^®^ group (*p* = 0.0495).

#### 3.3.5. Cathepsin K (CTSK)

**Figure 11:**
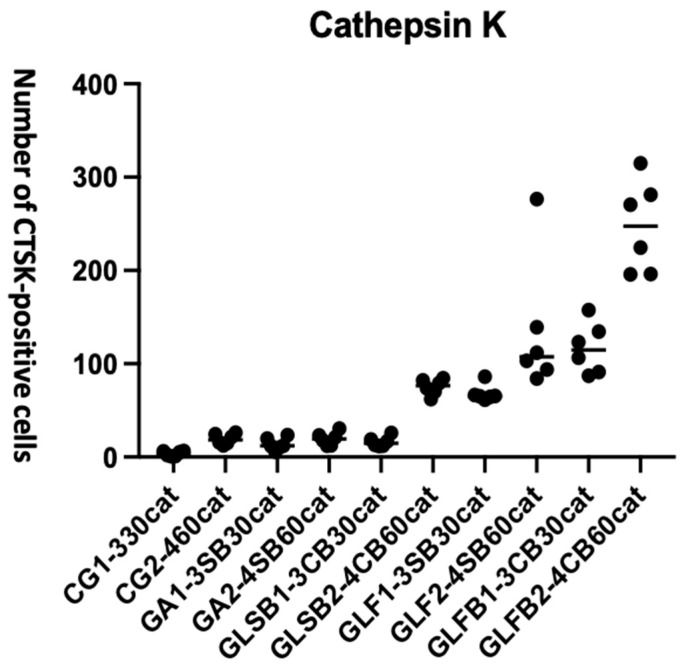
ANOVA analysis of CTSK-positive cells across the experimental groups. The *x*-axis represents the intervention groups at each time point, and the *y*-axis shows the number of CTSK-positive cells (0–400, in intervals of 100). PBM-FT exhibited the highest number of CTSK-positive cells compared to the other groups.

ANOVA results for CTSK: the number of positive cells resulted in significantly different between groups (F = 41.19, **** *p* < 0.0001) (Figure 11).

The details of the data of the groups’ comparisons are outlined in Table 7. PBM-FT with and without Bio-Oss^®^ significantly increased the CTSK antibody number both at 30 (*** *p* < 0.0001) and 60 days (**** *p* < 0.0001) compared to PBM-ST groups and control groups.

PBM-FT without Bio-Oss^®^ resulted in better outcomes than PBM-ST with Bio-Oss^®^ (*p* = 0.0214). and without (*p* = 0.0416). Moreover, the performance in terms of antibody-positive cells increased at 60 days (**** *p* < 0.0001). The presence of Bio-Oss^®^ increased the number of CTSK antibodies from 30 to 60 days, both in PBM-ST (*p* = 0.0221) and PBM-FT groups (**** *p* < 0.0001).

At 60 days, the laser-ST with Bio-Oss^®^ gave a better performance compared to PBM-ST without Bio-Oss^®^ (*p* = 0.0345); a higher significant difference was observed for the PBM-FT (**** *p* < 0.0001).

## 4. Discussion

The present study provides compelling evidence that PBM therapy significantly enhances bone regeneration in CSDs, particularly when combined with the osteoconductive scaffold Bio-Oss^®^.

Among all experimental conditions, the PBM-FT + Bio-Oss^®^ group exhibited the greatest extent of bone neoformation, indicating a clear synergistic interaction between 980 nm photonic energy delivered with FT and the scaffold.

Compared with untreated defects and those treated with Bio-Oss^®^ alone, laser PBM-treated sites showed accelerated defect closure, increased extracellular matrix deposition, and robust osteogenic activation, as evidenced by elevated expression of Runx2 and OCN. These findings are consistent with previous reports demonstrating the limited intrinsic regenerative capacity of spontaneous CSD healing and the slow early remodeling of Bio-Oss^®^ when used alone [66,82]. Collectively, the present results highlight that optimal bone regeneration requires both a suitable osteoconductive matrix and a biophysical stimulus, PBM, capable of achieving resident and recruited cells.

Notably, the FT delivery system is designed to provide a spatially uniform irradiance, which appears to be a critical determinant of therapy efficacy. Homogenous distribution across the defect likely optimizes cellular stimulation minimizing the risk of localized over- or under-dosage, a known limitation in PBM studies employing ST beam profiles [28,83,84].

### 4.1. Histological and IHC Evidence of Enhanced Bone Regeneration

Integrating histological and immunohistochemical analyses provides converging evidence that 980-nm PBM, particularly with an FT handpiece, accelerates calvarial bone healing at structural, cellular, and molecular levels. Across all staining modalities (H&E, MT, and IHC markers), PBM-FT combined with Bio-Oss^®^ consistently outperformed PBM-ST with or without Bio-Oss^®^, untreated controls, and Bio-Oss^®^ alone.

Immunostaining for Gli1, Runx2, OCN, CD34, and CTSK revealed that PBM-FT + Bio-Oss^®^ elicited the most pronounced regenerative response, indicating enhanced progenitor activation, osteogenic differentiation, angiogenesis, and remodeling. These findings suggest that PBM-FT induces. These findings indicate that PBM-FT + Bio-Oss^®^ synergistically promotes bone regeneration through early and sustained activation of tissue-repair pathways essential for effective bone regeneration.

#### 4.1.1. Histological Findings: Tissue Organization and Bone Matrix Maturation

Histological evaluation at both 30 and 60 days revealed marked differences among treatment groups. Untreated defects showed minimal intrinsic healing, characterized by persistent fibrous connective tissue, disorganized collagen architecture, and ongoing inflammatory infiltrate. Defects treated with Bio-Oss^®^ alone demonstrated partial bridging; however, large volumes of unremodelled graft particles remained, consistent with the known slow response kinetics of this xenograft [85].

Both PBM-ST and PBM-FT enhanced tissue regeneration relative to controls, with PBM-ST prompting improved early granulation tissue formation, vascularization, and moderate collagen organization. In contrast, PBM-FT produced more advanced regeneration, including dense and uniform organized collagen bundles, reduced inflammation, and more continuous periosteal and epithelial layers. These findings align with previous reports indicating that uniform energy delivery of PBM-FT improves dose precision and biological predictability.

The most advanced tissue maturation was observed in the PBM-FT + Bio-Oss^®^ group, which demonstrated extensive mineralized tissue formation and near-complete detect restoration by 60 days, strongly supporting a synergistic interaction between PBM and the scaffold.

#### 4.1.2. MT: Transition from Early Osteoid to Mature Bone

MT staining corroborated the H&E findings. Untreated and Bio-Oss^®^-only groups exhibited sparse collagen disposition and limited osteoid formation, whereas PBM (ST or FT)-treated defects showed dense collagen bundles and increased mineralized matrix, but the most uniform collagen distribution was observed in the PBM-FT group.

The PBM-FT + Bio-Oss^®^ group demonstrated the highest proportion of mature bone by 60 days, confirming the synergic effects of FT-PBM with the scaffold in accelerating osteoid maturation and accelerating mineralization.

#### 4.1.3. IHC: Molecular Mechanisms Supporting Enhanced Regeneration

IHC analyses further elucidated the molecular basis underlying the enhanced regenerative response.

- GLi1, a marker of progenitor cell activation, was significantly upregulated in PBM-treated groups, with maximal expression in the PBM-FT + Bio-Oss^®^, demonstrating strong early activation of osteoprogenitors. This is consistent with PBM-mediated activation of Hedgehog/GLi1 signaling, which plays a key role in skeletal progenitor proliferation and differentiation [86].
- Runx2 expression was elevated at 30 days across the PBM-treated groups, indicating enhanced early osteogenic differentiation, and remained high at 60 days in the PBM-FT + Bio-Oss^®^ group, reflecting sustained osteoblast activity. PBM photonic energy is known to be absorbed by mitochondrial cytochrome c oxidase, leading to an increase in ATP synthesis and activating signaling pathways such as Wnt/β-catenin and ERK, which promote osteoblast proliferation and Runx2-mediated differentiation [16].
- OCN expression increased markedly by 60 days in PBM-treated groups, consistent with advanced mineralization and osteoblast maturation. Mechanistically, 980-nm PBM stimulates mitochondrial activity, increasing ATP and low-level ROS, which activate PI3K/Akt and Wnt/BMP signaling pathways. This promotes osteogenic differentiation and upregulates Runx2 and OCN, supporting osteoblast maturation and mineralization [16].
- CD34 expression was strongest in PBM-FT-treated groups, indicating angiogenesis, a prerequisite for effective bone regeneration. PBM has been reported to prompt endothelial progenitor proliferation and vascularization through modulation of ROS and vascular endothelial growth factor (VEGF) signaling [87].
- CTSK, a marker of osteoclast-mediated remodeling, was increased in PBM-treated groups, suggesting active scaffold integration and bone turnover [88].

Together, these findings indicate that 980 nm PBM orchestrates a coordinated response by activating progenitor cells, enhancing osteogenic differentiation, stimulating angiogenesis, and promoting remodeling. This mechanistic evidence supports the synergetic effects of PBM-FT (homogenesis distribution) combined with Bio-Oss^®^.

### 4.2. Synergistic Mechanisms of PBM and Bio-Oss^®^

The enhanced osteogenesis observed with the combined application of photobiomodulation (PBM) and Bio-Oss^®^ likely arises from complementary biophysical and biological mechanisms. Bio-Oss^®^, a low-crystallinity bovine hydroxyapatite, exhibits a highly porous and micro-rough architecture, as demonstrated by physicochemical characterization studies of deproteinized bovine bone mineral [89]. Such microstructural heterogeneity introduces refractive index mismatches at the micrometer scale, which are known to promote multiple scattering of near-infrared (NIR) light in mineralized and porous biological materials [90]. This scattering-dominated light propagation may increase photon path length and local photon residence time within the grafted defect, thereby elevating the effective PBM dose received by osteogenic cells located at the Bio-Oss^®^–tissue interface.

At the cellular level, PBM photonic energy is absorbed by mitochondrial cytochrome-c oxidase activity, leading to a cascade of cellular and molecular activities, increasing ATP production, enhancing redox signaling, and modulating transcription factors involved in tissue repair [91,92,93]. These effects promote osteoblast proliferation and differentiation [94], upregulate angiogenic mediators such as VEGF and inflammatory responses, and collectively accelerate bone formation.

At the biomaterial interface, PBM may enhance protein adsorption (i.e., fibronectin and vitronectin) and modulate surface energy or local ionic microenvironments, thereby improving osteoblast adhesion and differentiation on Bio-Oss^®^. Furthermore, PBM-induced angiogenesis may compensate for the limited intrinsic vascular induction of slowly resorbing scaffolds, facilitating deeper cell infiltration and more effective remodeling.

These synergistic interactions provide a coherent mechanistic explanation for the enhanced bone formation observed and are consistent with previous studies reporting osteogenesis when PBM is combined with hydroxyapatite-based materials [95,96,97,98].

980 nm PBM laser light accelerates the healing process by influencing the water-containing tissues around Bio-Oss^®^. The light is absorbed by water in these tissues, increasing cellular activity (including osteoblasts) and enhancing vascularization. These effects work synergistically, promoting faster osteogenesis, improving graft integration with neoformation bone, and creating a better healing environment for the Bio-Oss^®^, crucial for successful bone regeneration.

The dosimetry chosen for this study, based on the pilot study, was measured to maintain the temperature between 25 and 28 °C ensure that optimal irradiance reached the target surface area, which proved to enhance the synergistic effects of the PBM light and improve the integration of the Bio-Oss^®^ graft into the neoformation bone.

### 4.3. Persistence of Bio-Oss^®^ During PBM-Enhanced Osteogenesis

Consistent with prior clinical and preclinical reports, Bio-Oss^®^ particles persisted throughout the study period, remaining embedded within newly formed bone [11,12]. Importantly, PBM did not accelerate scaffold resorption but instead prompted bone formation around and between particles. This indicated that PBM enhances cellular activity without compromising scaffold stability, reinforcing a synergetic rather than competitive interaction.

### 4.4. Novel 980-nm PBM Dosimetry Protocol Reaching the Target Surface Area

The PBM dosimetry protocol was precisely implemented to ensure that therapeutically relevant 980 nm laser energy reached the defect site despite attenuation by overlying tissues and biomaterials. This optimized energy delivery was achieved through direct optical quantification and careful control of beam delivery, allowing mechanistic interpretation of the biological outcomes. The enhanced osteogenic responses observed, including increased new bone formation, improved collagen organization, and accelerated defect bridging, highlight the importance of both delivered dose and spatial homogeneity in PBM-assisted bone regeneration.

Although the nominal laser power was identical for FT and ST beams (0.6 W), power meter measurements revealed substantial differences in the effective power delivered to the target area. Prior to tissue interaction, the applied power measured at the surface was 0.55 W for FT compared with 0.253 W for ST, indicating that the beam profile intrinsically influenced how much of the nominal power reached the effective irradiation area. This difference reflects the uniform spatial intensity distribution of the FT beam versus the centrally peaked Gaussian distribution of the ST beam, which distributes a significant fraction of energy outside the effective target region.

Using theoretical values of peak power, it is noted that nearly identical irradiance and fluence are applied by both handpieces to tissues at the center of the CSD, while those on the periphery only receive about 13.5% of that same energy [76,77,78,79]. This explains differences in PBM enhanced healing and the importance of using a flattop handpiece with predictable output.

After transmission through mouse skin, the measured target-surface average irradiance was 1.244 W/cm^2^ for FT and 0.487 W/cm^2^ for ST under shaved-skin conditions, and 1.131 W/cm^2^ for FT versus 0.413 W/cm^2^ for ST in the presence of Bio-Oss^®^ (Table 3). Analysis of power attenuation demonstrated that tissue-related losses were broadly comparable between beam types (approximately 59–60% for FT and 62–68% for ST), indicating that the optical properties of skin and biomaterials attenuated both beams similarly in relative terms. Consequently, the higher absolute irradiance delivered by FT at the defect surface primarily reflects its higher applied power and uniform spatial distribution rather than preferential transmission through tissue.

Because PBM effects are strongly dose-dependent, the superior biological responses observed in FT-treated groups are best explained by the combined effects of higher delivered dose and spatially uniform energy distribution, rather than beam profile alone. The FT beam minimized localized under-dosage across the defect surface, whereas the ST beam, despite exhibiting a higher central peak, delivered lower average irradiance due to peripheral intensity fall-off. This lower average dose likely limited the overall PBM efficacy of the ST beam.

These dosimetry differences corresponded with the enhanced osteogenic activity observed in histological and immunohistochemical analyses. The FT-PBM + Bio-Oss^®^ group exhibited increased new bone formation, improved collagen organization, and accelerated defect bridging. Molecular markers including Gli1, Runx2, OCN, CD34, and CTSK were significantly upregulated, indicating activation of progenitor cells, osteoblast differentiation, angiogenesis, and bone remodeling. The Bio-Oss^®^ scaffold further supported cell attachment, proliferation, and differentiation, synergizing with the higher and more uniformly distributed PBM dose delivered by the FT beam. In contrast, the ST-PBM and ST-PBM + Bio-Oss^®^ groups demonstrated less pronounced osteogenic activity, underscoring the necessity of both sufficient dose and spatial homogeneity for optimal regenerative outcomes.

Collectively, these findings demonstrate that optimizing both delivered dose and spatial uniformity is critical for achieving clinically meaningful bone regeneration. The FT beam’s ability to deliver consistent and sufficient irradiance across the defect surface provides a mechanistic explanation for the superior structural, cellular, and molecular outcomes observed, supporting a clinically translatable strategy for precise PBM-assisted bone regeneration.

The three-times-weekly treatment regimen was selected to maintain mitochondrial and osteogenic activation within an optimal therapeutic window (48 h interval) while avoiding photoinhibition, consistent with prior in vitro findings [28]. Together, direct optical validation, uniform FT beam delivery, and an appropriately timed treatment schedule underpin the superior regenerative outcomes observed and establish a reproducible PBM dosimetry framework. Infrared thermography further confirmed that skin surface temperature at the defect site remained stable between 25 and 28 °C during irradiation, with no measurable increase attributable to laser exposure, confirming that the bone regenerative effects are mediated by PBM without thermal contribution.

### 4.5. Novelty and Clinical Significance

This study is the first to systematically demonstrate and mechanistically explain the synergistic osteogenic effects of 980-nm PBM combined with Bio-Oss^®^ while directly quantifying the irradiance reaching the target surface. The multi-arm experimental design, including FT- and ST-delivered PBM, Bio-Oss^®^ alone, and combined treatments, enabled differentiation between isolated and synergistic effects.

Importantly, the findings demonstrate that PBM dose, in addition to beam spatial distribution, is a key determinant of biological outcome. Despite identical nominal laser power settings, FT-PBM consistently delivered higher average irradiance to the defect surface due to intrinsic beam delivery characteristics, resulting in stronger and more uniform regenerative responses. In contrast, ST-PBM delivered lower average doses, leading to comparatively weaker osteogenic outcomes. Thus, the superior effects observed with FT-PBM reflect the combined influence of sufficient delivered dose and spatial homogeneity rather than beam profile alone.

These results underscore the translational relevance of rigorously controlled PBM dosimetry. By integrating direct irradiance measurements with scaffold-assisted therapy, this study provides a framework for precise, reproducible, and clinically meaningful PBM-assisted bone regeneration.

The comparison between FT and ST beam profiles, particularly in combination with Bio-Oss^®^, underscores the importance of spatial energy distribution in PBM-assisted bone regeneration. FT consistently delivered higher and more uniform irradiance across the defect surface, resulting in superior bone formation, collagen organization, and upregulation of osteogenic markers compared with ST. This effect was especially pronounced when FT was combined with Bio-Oss^®^, indicating a synergistic interaction between uniform photonic energy and the osteoconductive scaffold. Clinically, these findings suggest that selecting a uniform beam profile may improve the predictability and efficacy of PBM in craniofacial bone repair, minimizing localized under- or overdosing and enhancing scaffold integration.

From a clinical perspective, these differences could translate into accelerated bone healing, allowing earlier functional rehabilitation and restoration of craniofacial integrity. Patients may experience shorter recovery times, reduced postoperative complications, and improved overall outcomes when FT-PBM is used in conjunction with grafting materials. Additionally, by improving the predictability and consistency of bone regeneration, FT-PBM could reduce the need for repeat surgeries or prolonged interventions, potentially lowering healthcare costs and increasing patient satisfaction. This emphasizes that precise control of beam profile and irradiance is not just a preclinical concern but has direct implications for translational therapy and clinical decision-making.

The clinician who sets laser output and then believes they are delivering comparable PBM therapy with either handpiece is incorrect. For PBM to have true value in enhancing hard tissue wound repair, all parameters and equipment must be diligently controlled.

The ex vivo pilot study demonstrated that light transmission through Bio-Oss^®^ and Bio-Gide^®^ within the defect was negligible (Table 3), indicating that these biomaterials do not significantly attenuate PBM energy. Because measurements were performed after creation of the CSD, intact calvarial bone was not part of the optical pathway. Laser light, delivered with FT or ST, was primarily attenuated by the overlying shaved mouse skin, with or without biomaterials. While mouse skin is thinner than human skin or oral mucosa, these results support the feasibility of PBM delivery in defect sites. They also highlight that irradiation parameters would require adjustment to account for increased tissue thickness in clinical translation.

### 4.6. Study Limitations and Future Directions

Despite the strengths of this study, several limitations should be acknowledged. The observation period was limited to 60 days, which allowed evaluation of advanced remodeling; however, longer-term studies are needed to assess the durability and functional stability of the regenerated bone. Incorporating micro-CT imaging alongside histological and immunohistochemical analyses would provide a more quantitative assessment of bone volume and structural regeneration.

Although nominal laser power was identical for FT and ST beams, power meter measurements demonstrated that the effective power and irradiance delivered to the target surface differed substantially due to intrinsic beam characteristics and subsequent tissue and scaffold attenuation.

While relative attenuation through skin and biomaterials was broadly comparable between beam types, differences in applied power resulted in persistent dose disparities at the defect site. Consequently, the observed biological differences reflect the combined effects of beam delivery characteristics and resulting dose.

Future studies could aim to normalize delivered dose across beam profiles, although achieving equivalent spatial and energetic distributions remains technically challenging. Such approaches would further clarify the isolated contribution of beam profile to PBM efficacy. Additionally, future research should focus on:

- Translation to randomized clinical trials evaluating safety and efficacy in humans;
- Personalized PBM dosimetry accounting for patient-specific optical properties;
- Long-term functional and mechanical outcomes relevant to clinical practice.

## 5. Conclusions

This preclinical translational study, for the first time, employed a standardized PBM irradiation protocol that ensured effective delivery of therapeutic light to the CSD (target) surface area.

The results demonstrate that PBM therapy, particularly when combined with Bio-Oss^®^ and delivered via the FT handpiece, significantly accelerates both early- and late-stage osteogenesis. Enhanced expression of key osteogenic markers, improved matrix deposition, and more advanced bone remodeling highlight the synergistic interaction between photobiomodulation and the osteoconductive scaffold. These findings provide a strong mechanistic and histological rationale for the clinical translation of PBM-assisted bone regeneration.

Future studies should build on this evidence by conducting well-designed RCTs in humans to validate the efficacy, optimal dosimetry, and clinical applicability of PBM combined with biomaterial scaffolds. Such trials could pave the way for standardized protocols in regenerative dentistry and craniofacial bone repair, ultimately translating preclinical benefits into predictable clinical outcomes.

## Figures and Tables

**Table 1:** Allocation of mice to experimental groups and euthanasia time points. A total of 120 mice were divided into twelve independent groups (10 mice per group). Each treatment condition was subdivided into two subgroups, which were euthanized either 30 or 60 days after treatment. Each animal was evaluated at a single time point. Created by the authors.

Experimental Groups	30 Days Post-Treatment	60 Days Post-Treatment
Only CSD (control)	CG1	CG2
Bio-Oss^®^ alone (control)	CG3	CG4
PBM-ST	GA1	GA2
PBM-FT	GLF1	GLF2
PBM-ST + Bio-Oss^®^	GLSB1	GLSB2
PBM-FT + Bio-Oss^®^	GLFB1	GLFB2

**Table 2:** Device specifications, main therapeutic power output measured using a power meter, actual therapeutic power delivered through the 0.5-cm ring after accounting for 10% glass plate absorption (measured by power meter), and calculated values of the associated parameters for both FT and ST. Created by the authors.

Device Specifications	Manufacturer	Lambda-Doctor Smile	
	Model Identifier	GaAIAs	
	Probe design	Wiser 2	
	Emitters type	Class IV	
	Device classification	Diode laser	
	Probe Design	Single	
	Polarization	Linear	
	Operating emission mode	CW	
	Spot diameter (cm)	ST: 0.83	FT: ~1
	Beam area (cm^2^)	ST: 0.50	FT: 0.785
	Beam diverange (°)	ST: 8.85 (1/2 angle)	FT: 0
PBM Dosimetry	Wavelength (nm)	980	
	Nominal power output (W)—set on laser interface	ST: 0.6	FT: 0.6
	Main therapeutic power output (W) measured with power meter	ST: 0.42	FT: 0.6
	Actual therapeutic power output (W) measured with power meter through masking ring, after adding the 10% value of glass plate absorption	ST: 0.253	FT: 0.55
	Energy (J) per session at therapeutic power output	ST: 15.2	FT: 0.33
	Irradiance (W/cm^2^) per session at therapeutic power output	ST: 1.289	FT: 2.801
	Fluence (J/cm^2^) per session at therapeutic power output	ST: 77.3	FT: 168.1
	Total energy (J) (total 6 sessions) at therapeutic power output	ST: 463.9	FT: 1008.4
Treatment protocol	Number of irradiated spots	1	
	Irradiation time (s)	60	
	Treatment duration	Two consecutive weeks	
	Total treatment sessions	6	
	Irradiation frequency	Three-weekly	
	Light-shaved skin distance	In contact	
	Treatment technique	Stationary/ Spot technique	

**Table 3:** PBM dosimetry reaching the target surface area (CSD) under different experimental conditions: no shaved skin or biomaterials, and shaved skin without biomaterials, shaved skin with Bio-Gide^®^ + Bio-Oss^®^. The reported area (0.1963 cm^2^) corresponds to the effective irradiated target surface defined by the defect and does not represent the nominal beam cross-section. All the values of the power outputs were measured by the power meter. Created by the authors.

Optical Condition	Effective Radiated Surface Area (0.1963 cm^2^)	Therapeutic Power (W)	Energy (J)/Point	Average Irradiance (W/cm^2^)	Average Fluence (J/cm^2^)	Peak Irradiance (W/cm^2^)	Peak Fluence (J/cm^2^)
No shaved mouse skin and no biomaterials	FT ST	0.55 0.253	33.0 15.2	2.80 1.289	168.1 77.3	2.80 2.578	168.1 154.6
Shaved mouse skin and no biomaterials	FT	0.244	14.64	1.224	74.6	1.244	74.6
	ST	0.0956	5.74	0.487	29.2	0.974	58.4
Shaved mouse skin and Bio-Gide^®^ + Bio-Oss^®^	FT	0.222	13.32	1.131	67.8	1.131	67.8
	ST	0.0811	4.87	0.413	24.8	0.826	49.6

**Note: All powers and dosimetry values are corrected for ~10% transmission loss through the supporting glass plate.**

**Table 4:** Outlines the quantitative data of multiple comparative groups using the Holm-Šídák’s test to evaluate the CD34 positive cells. Significance notation: * *p* < 0.05; *** *p* < 0.001; **** *p* < 0.0001.

Comparative Groups	Mean Diff.	Summary	Adjusted *p*-Value
CG1 vs. GLF1	−79.67	****	<0.0001
CG1 vs. GLFB1	−114.2	****	<0.0001
GA1 vs. GLF1	−65.30	****	<0.0001
GA1 vs. GLFB1	−99.83	****	<0.0001
GLSB1 vs. GLF1	−63.90	****	<0.0001
GLSB1 vs. GLFB1	−98.43	****	<0.0001
GLF1 vs. GLFB1	−34.53	*	0.0458
CG2 vs. GLSB2	−62.40	****	<0.0001
CG2 vs. GLF2	−89.63	****	<0.0001
CG2 vs. GLFB2	−274.4	****	<0.0001
GA2 vs. GLSB2	−56.20	***	0.0001
GA2 vs. GLF2	−83.43	****	<0.0001
GA2 vs. GLFB2	−268.2	****	<0.0001
GLSB2 vs. GLFB2	−212.0	****	<0.0001
GLF2 vs. GLFB2	−184.8	****	<0.0001
GA2 vs. GLF1	−60.10	****	<0.0001
GA2 vs. GLFB1	−94.63	****	<0.0001
GLSB2 vs. GLFB1	−38.43	*	0.0178

**Table 5:** Outlines the quantitative data of multiple comparative groups using the Holm-Šídák’s test to evaluate the GLi1 positive cells. Significance notation: * *p* < 0.05; ** *p* < 0.01; *** *p* < 0.001; **** *p* < 0.0001.

Comparative Groups	Mean Diff.	Summary	Adjusted *p* Value
CG1 vs. GLF1	−70.73	**	0.0062
CG1 vs. GLFB1	−102.2	****	<0.0001
GA1 vs. GLF1	−61.47	*	0.0279
GA1 vs. GLFB1	−92.90	***	0.0001
GLSB1 vs. GLFB1	−86.23	***	0.0004
CG2 vs. GLSB2	−57.27	*	0.0452
CG2 vs. GLF2	−117.0	****	<0.0001
CG2 vs. GLFB2	−251.1	****	<0.0001
GA2 vs. GLF2	−114.2	****	<0.0001
GA2 vs. GLFB2	−248.3	****	<0.0001
GLSB2 vs. GLF2	−59.77	*	0.0338
GLSB2 vs. GLFB2	−193.8	****	<0.0001
GLF2 vs. GLFB2	−134.1	****	<0.0001
GA2 vs. GLFB1	−85.97	***	0.0004
GLF1 vs. GLF2	−59.67	*	0.0338
GLFB1 vs. GLFB2	−162.3	****	<0.0001

**Table 6:** Pairwise comparison of osteocalcin-positive cell counts between treatment groups using Holm–Šídák’s multiple comparisons test. Adjusted *p*-values were calculated using Holm–Šídák’s method. Significance notation: * *p* < 0.05; ** *p* < 0.01; **** *p* < 0.0001.

Comparative Groups	Mean Diff.	Summary	Adjusted *p*-Value
CG1 vs. GLF1	−69.23	**	0.0078
CG1 vs. GLFB1	−124.7	****	<0.0001
CG2 vs. GLF2	−105.4	****	<0.0001
CG2 vs. GLFB2	−225.9	****	<0.0001
GA1 vs. GLF1	−56.37	*	0.0495
GA1 vs. GLFB1	−111.8	****	<0.0001
GA2 vs. GLSB2	−56.80	*	0.0495
GA2 vs. GLF2	−103.0	****	<0.0001
GA2 vs. GLFB2	−223.5	****	<0.0001
GLSB1 vs. GLF1	−57.13	*	0.0495
GLSB1 vs. GLFB1	−112.6	****	<0.0001
GLSB1 vs. GLFB1	−166.7	****	<0.0001
GLSB2 vs. GLFB2	−166.7	****	<0.0001
GLF2 vs. GLFB2	−120.5	****	<0.0001

**Table 7:** Outlines the quantitative data of multiple comparative groups using the Holm-Šídák’s test to evaluate Cathepsin K (CTSK) positive cells. Significance notation: * *p* < 0.05; ** *p* < 0.01; **** *p* < 0.0001.

Comparative Groups	Mean Diff.	Summary	Adjusted *p* Value
CG1 vs. GLF1	−64.63	**	0.0091
CG1 vs. GLFB1	−113.0	****	<0.0001
GA1 vs. GLF1	−54.33	*	0.0416
GA1 vs. GLFB1	−102.7	****	<0.0001
GLSB1 vs. GLFB1	−100.2	****	<0.0001
GLSB1 vs. GLSB2	−58.93	*	0.0221
GLFB1 vs. GLFB2	−130.5	****	<0.0001
CG2 vs. GLF2	−115.4	****	<0.0001
CG2 vs. GLFB2	−227.8	****	<00001
GA2 vs. GLSB2	−55.80	*	0.0345
GA2 vs. GLF2	−115.2	****	<0.0001
GA2 vs. GLFB2	−227.6	****	<0.0001
GLSB2 vs. GLF2	−59.37	*	0.0214
GLSB2 vs. GLFB2	−171.8	****	<0.0001
GLF2- vs. GLFB2	−112.4	****	<0.0001

## Data Availability

All the data is included in the text.

## Supplementary Materials

The following supporting information can be downloaded at: https://www.mdpi.com/article/10.3390/jfb17030125/s1. Supplementary File S1: Completed ARRIVE 2.0 checklist for this study.
